# Analysis of urban land cover influence to organic carbon and nutrients in surface water via impacted groundwater

**DOI:** 10.1007/s10661-020-8095-7

**Published:** 2020-01-27

**Authors:** Katarzyna Puczko, Elżbieta Jekatierynczuk-Rudczyk

**Affiliations:** Department of Environmental Protection, Faculty of Biology, University of Bialystok, Ciołkowskiego 1J, 15-245 Białystok, Poland

**Keywords:** HERCULES model, Land cover, Nitrogen, Phosphorous, Surface water, Shallow groundwater

## Abstract

This paper presents an object-oriented approach for analysing and characterising the urban landscape structure and its influence on the quality of surface waters and shallow groundwater. We investigated springs, streams and ponds from an urban area. The land cover classification was adopted with the conceptual framework of urban land cover (HERCULES model). This study has demonstrated that water quality in the urban area is strongly related to land cover, and the degree of its transformation is not the same in all types of waters. The land with forests and shrubs does not have many extreme values in water chemical characteristics. Statistical analyses indicated that the main environmental factors influencing water chemistry are impermeable surfaces such as buildings. They are an essential element which deteriorates water quality. The patches with buildings and pavements were characterised by a wide gradient of nutrient concentration in rivers and ponds. Shallow groundwater had a limited effect on surface water quality.

## Introduction

Humans have experienced rapid urban expansion. More than half of the world population now lives in cities, and approximately 5% of global land has been converted to urban areas (Kopecká et al. 2018; Grimm et al. 2008; Lutz et al. 2001). The sustainability of an increasingly urbanised world is closely related to the structure of urban landscapes characterised by combinations of different land cover types (Pan et al. 2017). Although urban land cover constitutes only a small percentage of global land, it significantly alters climate, hydrology and biogeochemistry at local, regional and global scales (Kampffmeyer et al. 2018).

The relationship of land and water in urban areas is a complex of physical, ecological and social interactions (Cadenasso et al. 2008). Urban ecology treats an urban space as a heterogeneous ecological system (Willig and Scheiner 2011) with a combination of natural and engineered landscape elements (Pickett et al. 2013). Hybrid compositional elements of urban space with the proportion of vegetative cover, ground surfaces and built structures have been included in the High Ecological Resolution Classification for Urban Landscapes and Environmental Systems (Cadenasso et al. 2007). The model of urban space integrates built and natural components. It facilitates understanding the fine-scale structure of urban watersheds (Cadenasso et al. 2008). It is widely used in ecological research conducted in urban areas (Herrmann and Cadenasso 2017; McConaghie and Cadenasso 2016; Beck et al. 2016).

Urban hydrology is drastically modified when compared with agricultural and forested ecosystems (Paul and Meyer 2001; Walsh et al. 2005). Urban development creates large amounts of impervious surfaces for roads, parking, buildings and sidewalks. As impervious surfaces are created, large increases in runoff volume and rates occur (Alamdari et al. 2017). Streams integrate human disturbances across urban catchments. Their main role is transporting surface runoff. On the one hand, the hydrologic connectivity is increased via storm drains between impervious surfaces and streams. Channel incision causes decreased hydrologic connectivity between the stream and its catchment (Pickett et al. 2011). The actions of straightening the river channels and consolidating the bottom consequently lead to impoverishment and devastation of aquatic ecosystems and often cause irreversible changes in the water cycle. Urban rivers often lose their hydraulic contact with the groundwater. Rivers flowing with cemented bottom or a tube hidden in the ground do not fulfil their natural functions. The hydraulic parameters of urban rivers are different from those with natural river beds (Puczko and Jekatierynczuk-Rudczyk 2014). This ecological degradation of rivers in urban landscapes has been coined “urban stream syndrome” (Walsh et al. 2005; Meyer et al. 2005). The management of urban streams treats the river as a tube without groundwater exchange (Gabor et al. 2017, Walsh et al. 2005). It is considered that rivers are supplied with surface runoff and water quality depends mainly on surface contaminations (Goonetilleke et al. 2005, Burns et al. 2012). However, in some urban areas, the hydraulic relationship between groundwater and surface water still exists. Many studies have found evidence of urbanisation altering shallow groundwater quality (Cooper et al. 2014; Lawrence et al. 2013; Mayer et al. 2010).

Human activities have greatly altered biogeochemical cycles of many water chemical compounds. The nitrogen and phosphorus cycle, transformed by urbanisation processes, accelerates the rate of delivery of nutrients to surface water (Metson et al. 2017). The ability to predict transformations of nutrients, organic carbon and many other chemical water compounds often relies on small-scale catchment studies where detailed measurements can be made. Global scale models can abstract from real-world management options (Metson et al. 2017).

Therefore, for the proper management of water resources, including water quality, we need a better understanding of the impact of shallow groundwater to urban streams. In this study, we focused on two questions: “What is the intensity of urban land use impact on the groundwater and stream water concentrations of organic carbon, nitrogen and phosphorus forms in different urban land cover?” and “What is the effect of shallow groundwater to water quality in urbanised lotic and lentic aquatic systems?”

## Study area

Białystok is situated in the Białystok Upland of the Podlaskie Plain, part of what is known collectively as the Green Lungs of Poland. Near the city, there are many unique natural ensembles on a European scale. These include the Narew National Park—a swampy valley with moraine hills typical of a braided river, an abode of extremely rare species of water-marsh birds and the Knyszyn Forest Landscape Park—one of the best preserved forest complexes in Poland that contains varied postglacial sculpture, streams and springs. Białystok covers an area of 102 km^2^, characterised by the presence of moraine plateaus, the slopes of which are cut by numerous valleys.

Białystok city, with a population of over 280 thousand residents, ranks second place in terms of population density in Poland (2911 people per km^2^). Forests are an important part of the city and occupy around 1.756 ha (17.2% of the administrative area of the city). Białystok is the fifth most forested city in Poland. Parks and squares create a specific and healthy microclimate. Białystok as the first city in Poland was admitted to the European Healthy Cities Network, an international project of the World Health Organization.

The leading industries in the city’s economy are food processing, electrical engineering, machine industry, textiles and wood building materials. The composition of the districts varies from residential near the city centre, with a combination of multi-story apartment buildings and individual houses on small parcels, to industrial and agricultural at the city edges.

**Fig. 1:**
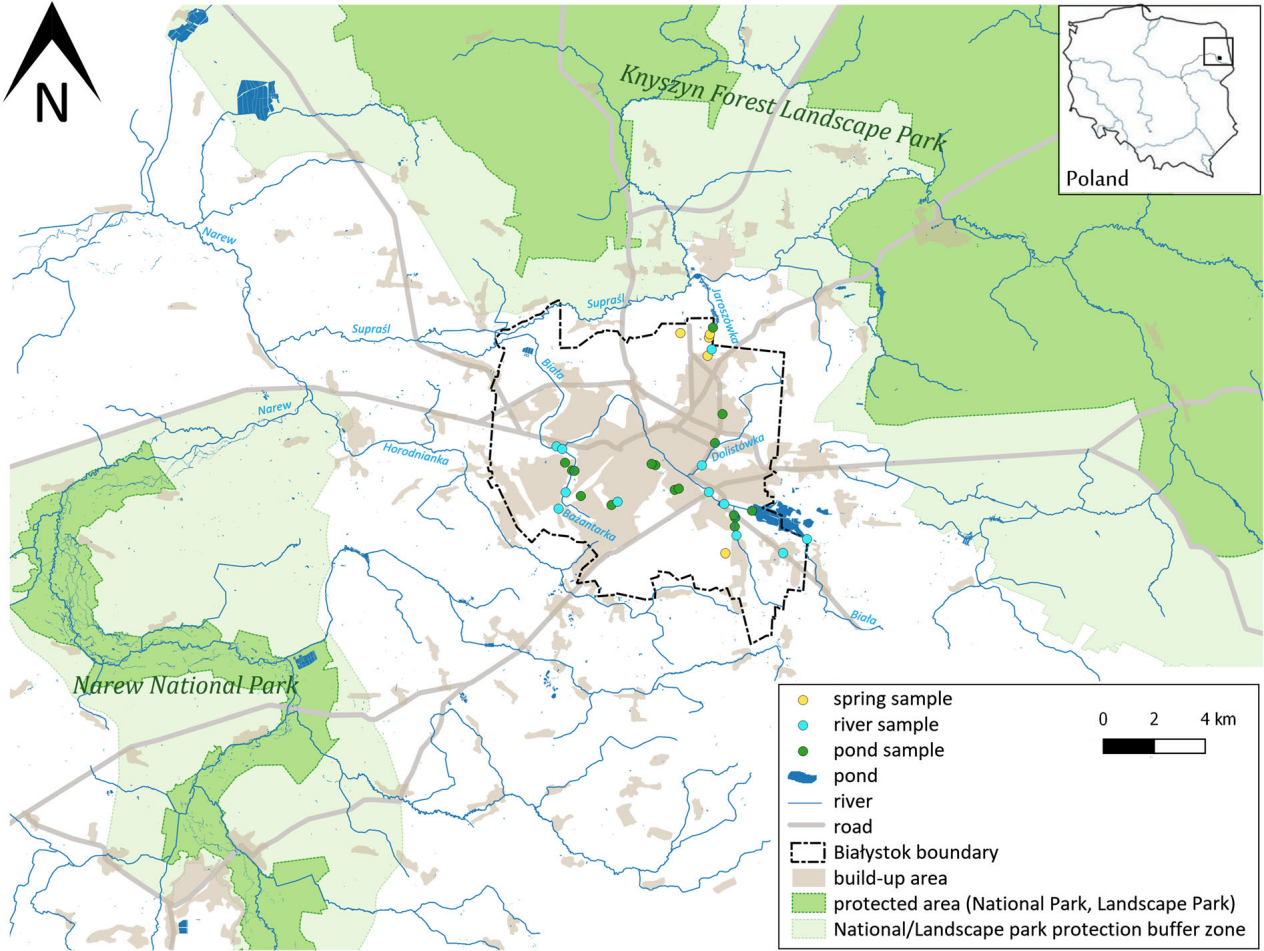
Map of sampling sites

Białystok area has a complex hydrographic network (Fig. 1), that owes its shape to varied terrain formed during the Weichselien glaciation. Almost the entire city lies in the catchment area of the Biała river, which is a left-bank tributary of the Supraśl river. Supraśl river drains only the northern edge of the city, whereas Horodnianka, which enters directly into the Narew river, drains the southern edge of Białystok. The storm water drainage system covers 47.35% of the city surface, and it is an important factor affecting river flow and water quality of streams (Kwiatkowski et al. 2004, Jekatierynczuk-Rudczyk 2008). A unique element in the landscape of the city is the springs. Natural outflows of groundwater are rare in the urban area, and its presence confirms a well-preserved natural environment (Puczko et al. 2018). Springs of Białystok are located mainly in forests and parks, with the exception of one limnocrene located near buildings. Springs with the average yield from 0.1 to 5.0 dm^3^/s are endangered due to leaks in the sewerage network and surface contamination. The development of the city and transportation network indirectly influences spring water quality (Puczko et al. 2018). Morphological and hydrological characteristics of springs, rivers and ponds in Białystok are presented in Tables 1, 2 and 3.

**Table 1:** Morphological and hydrological characteristics of springs in Białystok (Puczko et al. 2014)

	Geographical coordinates	Hydrological location	Q av. (dm^3^ s^−1^)	Type of spring	Surface of the niche (m^2^)	Geology of the vadose zone	Land use
S1	53° 05′ 49.3″ N	Biała catchment	0.5	Rheocrene	28	Sand, gravel, peat	Forest
	23° 11′ 59, 4″ E						
S2	53° 10′ 39.7″ N	Biała catchment	0.3	Rheocrene	500	Sand, gravel, peat	Forest
	23° 10′ 59.1″ E						
S3	53° 10′ 02.8″ N	Jaroszówka catchment	2.4	Artificial limnocrene	450	Sand, peat	urban area
	23° 11′ 49.7″ E						
S4	53° 10′ 38.8″ N	Jaroszówka catchment	5.2	Rheocrene	250	Sand, gravel	Forest
	23° 11′ 59.1″ E						
S5	53° 10′ 23.9″ N	Jaroszówka catchment	0.3	Rheocrene	125	Sand, gravel	Forest/grassland
	23° 11′ 52.1″ E

**Table 2:** Morphological and hydrological characteristics of rivers in Białystok (Kwiatkowski et al. 2004)

	Geographical coordinates	Name of river/stream	Length (km)	Average flow (dm^3^/s)	Catchment area (km^2^)	Urban catchment (%)
R1	53° 06′ 31.8″ N	Stream in Sobolewo	0.4			2
	23° 16′ 6.64″ E					
R2	53° 05′ 0.62″ N	Biała	29.4	1110	108	80
	23° 15′ 20.73″ E					
R3	53° 06′ 46.33″ N	Biała				
	23° 12′ 58.19″ E					
R4	53° 07′ 9.42″ N	Biała				
	23° 11′ 32.55″ E					
R5	53° 08′ 20.49″ N	Biała				
	23° 06′ 22.76″ E					
R6	53° 07′ 32.68″ N	Dolistówka	6.5	80	69	80
	23° 11′ 1.06″ E					
R7	53° 06′ 14.89″ N	Dojlidy stream	0.5			80
	23° 12′ 24.22″ E					
R8	53° 07′ 30.85″ N	Bażantarka	3.9	29	11.3	99
	23° 06′ 56.77″ E					
R9	53° 08′ 17.72″ N	Bażantarka				
	23° 06′ 25.92″ E					
R10	53° 07′ 0.28″ N	Stream (Pogodna street)	0.3			100
	23° 08′ 24.46″ E					
R11	53° 07′ 29.63″ N	Stream (Popiełuszki street)	1			99
	23° 06′ 37.28″ E					
R12	53° 10′ 49.88″ N	Jaroszówka	1	14	3.4	4
	23° 12′ 10.62″ E

**Table 3:** Morphological characteristics of ponds in Białystok and Carlson’s trophy state index (TSI) (E—eutrophy; H—hypertrophy) (Puczko et al. 2014)

	Geographical coordinates	Hydrological location	Surface (km^2^)	Trophy type acc. to TSI
P1	53° 06′ 21″ N; 23° 12′ 23″ E	Biała catchment	0.02	H
P2	53° 06′31.6″ N; 23° 13′ 20.9″ E	Biała catchment	0.25	E
P3	53° 06′ 34″ N; 23° 12′ 25.7″ E	Biała catchment	0.008	H
P4	53° 06′ 36.6″ N; 23° 12′ 26.1″ E	Biała catchment	0.001	H
P5	53° 07′ 14.5″ N; 23° 10′ 24.2″ E	Biała catchment	0.01	H
P6	53° 07′ 15.8″ N; 23° 10′ 30.8″ E	Biała catchment	0.01	H
P7	53° 07′ 37.7″ N; 23° 10′ 3.9″ E	Biała catchment	0.0006	H
P8	53° 07′ 40.1″ N; 23° 10′ 7.9″ E	Biała catchment	0.005	H
P9	53° 08′ 46.9″ N; 23° 12′ 11.3″ E	Biała catchment	0.008	E
P10	53° 08′ 10.5″ N; 23° 11′ 52.1″ E	Biała catchment	0.002	H
P11	53° 07′ 0.56″ N; 23° 8′ 6.23″ E	Bażantarka catchment	0.004	H
P12	53° 07′ 13.8″ N; 23° 7′ 2.53″ E	Bażantarka catchment	0.017	E
P13	53° 07′ 45.7″ N; 23° 6′ 53″ E	Bażantarka catchment	0.003	H
P14	53° 07′ 46.5″ N; 23° 6′ 48.9″ E	Bażantarka catchment	0.001	H
P15	53° 07′ 58″ N; 23° 5′ 52.8″ E	Bażantarka catchment	0.002	H
P16	53° 10′ 51.4″ N; 23° 12′ 11.2″ E	Jaroszówka catchment	0.008	H

## Methods

### Geospatial analysis

The land cover classification was adopted with the conceptual framework of urban land cover. It is known as High Ecological Resolution Classification for Urban Landscapes and Environmental Systems (HERCULES) (see Cadenasso et al. (2007) for a full description of the HERCULES land cover model). The HERCULES model focuses on land cover, not including land use. HERCULES incorporates vegetation structure and cover. It explains how the cover of land in urban areas is related to function. Spatial data can be added as a data layer if a specific research question requires it (Cadenasso et al. 2008). The model integrates built and natural components and helps clarify the structure of urban heterogeneity. An ecological approach facilitates understanding the fine structure of surface water networks in urban areas. The use of the HERCULES model allows us to study the impact of urbanisation on the natural environment, including water quality.

**Fig. 2:**
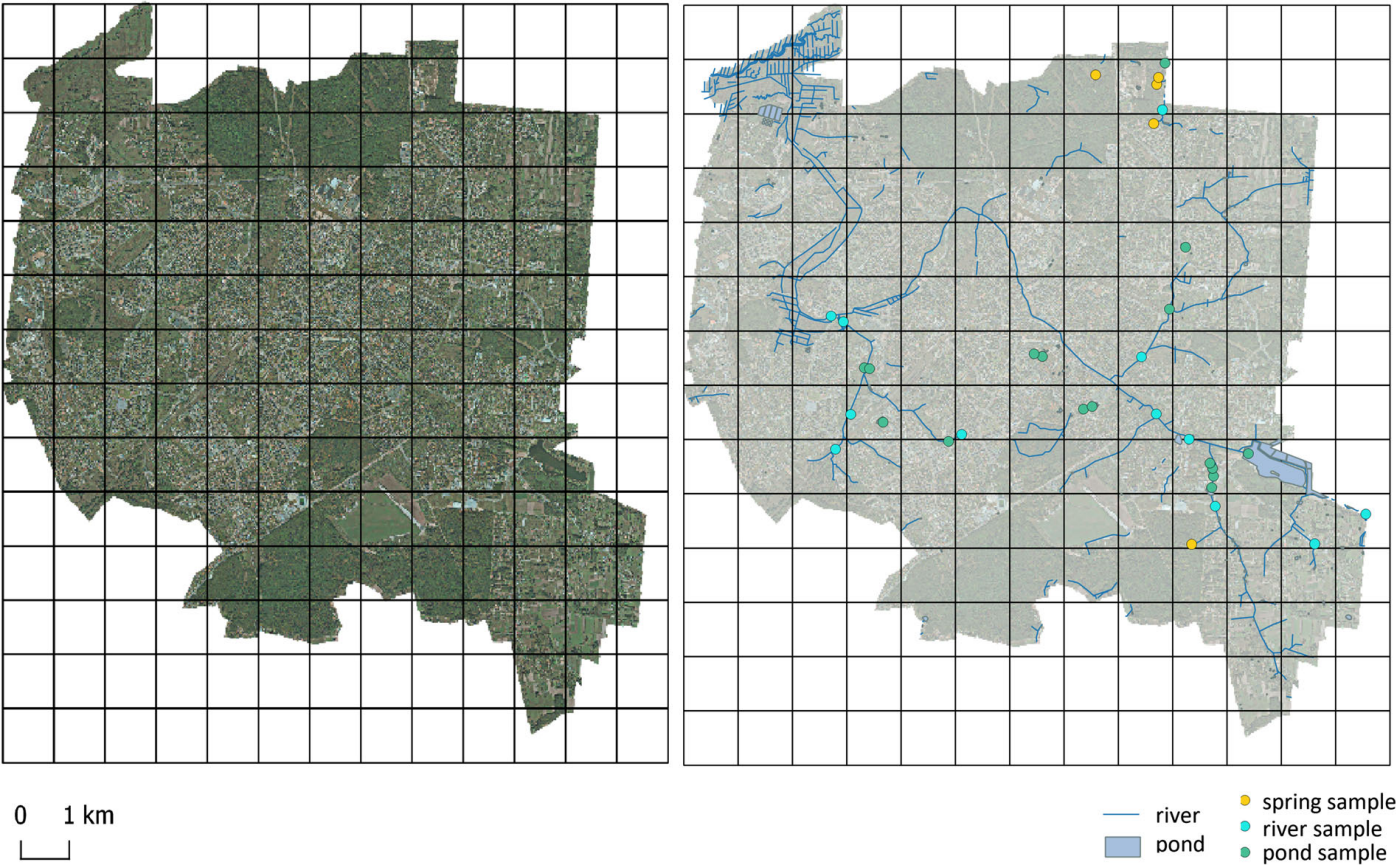
Patches of Białystok city used in HERCULES model

Six land cover features characterised by an urban land heterogeneity were described in the classification map: (1) coarse-textured vegetation with trees and shrubs (CV), (2) fine-textured vegetation with grasses and herbs (FV), (3) bare soil, (4) pavement, (5) buildings and (6) water (ponds, streams) (Cadenasso et al. 2008; Zhou et al. 2011; Zhou and Troy 2008). Hierarchy of urban landscape structure for the HERCULES model is presented in Fig. 2. The land cover classification map was derived from aerial imagery collected for the Białystok city in October 2014 (www.codgik.gov.pl/index.php/zasob/ortofotomapa.html, n.d.).

Measurements of the composition and configuration of land cover were made in each patch (a polygon layer). The land cover classification map and HERCULES model were used to describe the percentage share of land cover features in each patch, calculated in QGIS 2.18.13. The total number of patches in Białystok was 132, with a mean patch size of about 1 km^2^ (Fig. 2, Table 4). We calculated configuration metrics for buildings, pavement, CV and FV, bare soil and water. The proportional cover of coarse and fine vegetation, pavement and buildings is scored into 5 categories (0 = none; 1 = present < 10%; 2 = 11–35%; 3 = 36–75%; 4 = > 75%) (Zhou et al. 2011).

**Table 4:**
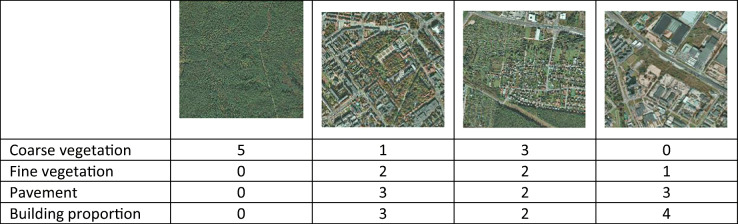
Examples of patches classified using HERCULES model

### Water quality data

**Fig. 3:**
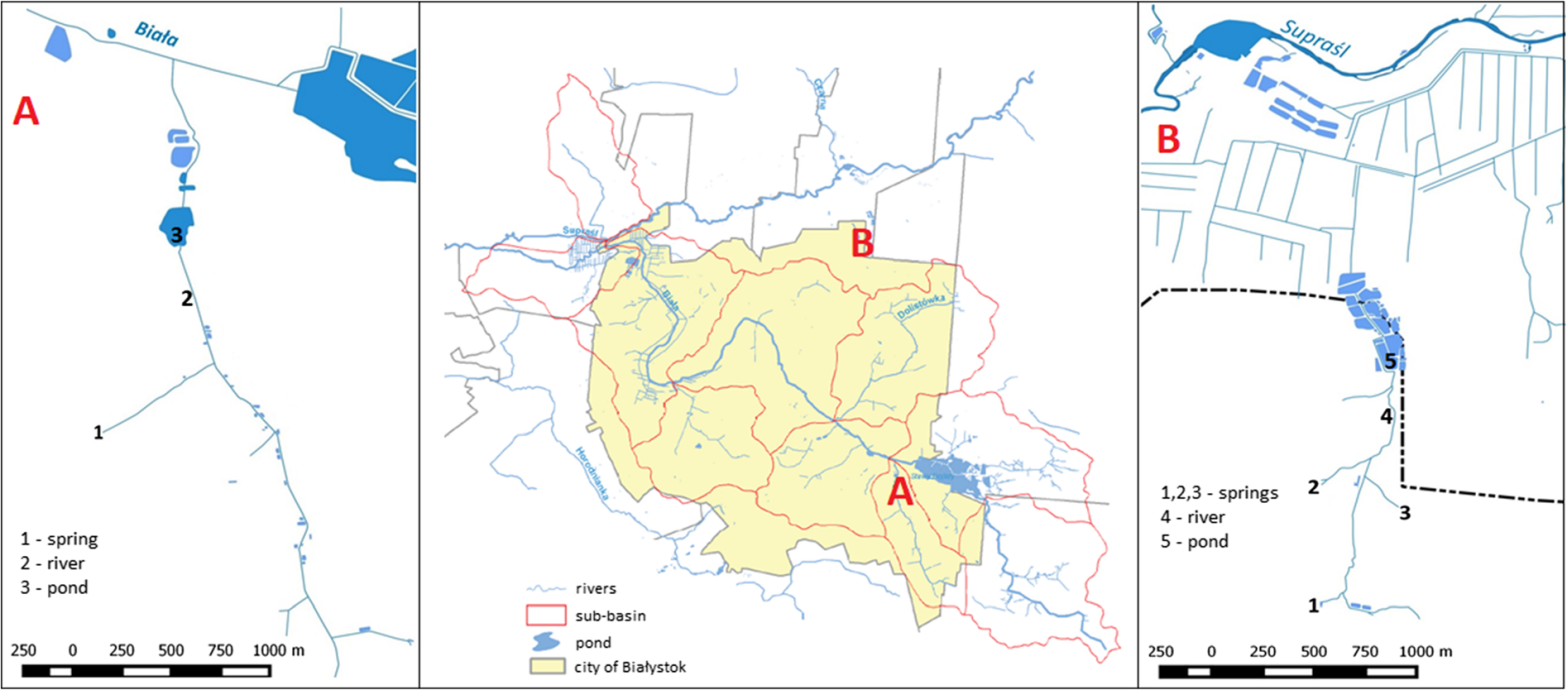
Location of sampling sites in Dojlidy stream catchment (A) and Jaroszówka catchment (B)

Water tests were conducted in water years 2013/2014 and 2014/2015. The study included 5 springs (S1–S5) (Table 1), 8 rivers (R1–R12) (Table 2) and 16 ponds (P1–P16) (Table 3). We collected samples in lower and upper parts of Bażantarka river (2 sampling sites) and Biała river (4 sampling sites). Furthermore, we conducted detailed research in the initial parts of rivers—spring, stream and pond in the Dojlidy stream catchment (Fig. 3, point A) and three springs, rivers and ponds in the Jaroszówka river catchment (Fig. 3, point B).

Water temperature, pH and electrolytic conductivity (EC) were determined with HachLange multiparameter probe (HQ40). Chemical water analyses were carried out in accordance with ISO standards, methods described by APHA (1998).

The following analyses were performed: ammonium nitrogen (NH_4_^+^-N) (indophenol blue colorimetry method), nitrate nitrogen (NO_3_^−^-N) (by reduction and colour development with sulphanilamide and N-(1-naphthyl)-ethylenediamine dihydrochloride) and nitrite nitrogen (NO_2_^−^-N) (sulphanilamide method) (APHA 1998). Kieldahl nitrogen was analysed by Tecator 2300 (Kjeldahl analyser). Mineral nitrogen (TIN) was calculated as the sum of NH_4_^+^-N, NO_3_^−^-N and NO_2_^−^-N. Total nitrogen (TN) was calculated as the sum of Kieldahl nitrogen and nitrites and nitrates. Phosphorus ions were determined by the molybdenum method measured according to standard methods (APHA 1998). Five phosphorus fractions were distinguished: total fraction (TP), dissolved fraction (DP), soluble reactive fraction (SRP), dissolved organic fraction (DOP) and particle fraction (PP). Total fraction was determined in non-filtered water after mineralisation. The soluble fraction was determined in water filtered through a filter GF/F with a pore diameter of 0.45 μm and mineralisation. The reactive fraction was determined in water filtered through a filter GF/F without prior mineralisation. HCO_3_^−^ ions were determined by titration with hydrochloric acid (HCl) in the presence of an indicator—methyl orange (APHA 1998). Dissolved organic carbon concentration (DOC) was determined by Shimadzu TOC-5050A analyser with an IR detector.

### Statistical analysis

A variety of statistical models have been used to explore the relationship between organic carbon, phosphorous and nitrogen management in shallow groundwater and surface water. Average annual concentration of DOC and nutrients were calculated using the seasonal data available for each of the 33 sample sites for the analysis period.

The relationships between landscape variables and water quality of surface water and shallow groundwater were performed using similar statistical analyses. The normality of distribution of variables was tested using the Kolmogorov-Smirnov goodness of fit test. The Kruskal-Wallis test was used to determine whether there was a significant difference between water quality in different types of water bodies. The statistical significance level was set at *p* = 0.01.

Standardised coefficients of variation ($Cv=\left(\frac{\mathrm{SD}}{x}\right)\times 100\%$); where SD is the standard deviation and *x* is the sample mean) were calculated to compare the variability of water quality parameters in different types of water bodies.

Spearman’s rank correlation analysis was used to show the relationships among water quality variables, land cover features and water quality. The statistical significance level was set at *p* = 0.05 and *p* = 0.01.

The nutrients and DOC concentration assigned to different water types was compared among sampling sites using the analysis of variance (ANOVA), and Tukey’s HSD (honestly significant difference) test was carried out to determine in which sites significant differences occurred.

Principal component analysis (PCA) was used to visually examine and compare differences in water quality between groundwater, streams and ponds. PCA is a multivariate statistical method which is applied in environmental studies to explain data structures (Vialle et al. 2011). PCA indicates the most meaningful parameters which describe the whole data set interpretation with minimal loss of original information (Helena et al. 2000).

To reduce the deviation from normality, landscape data (predictors) and water quality data (response variables) were transformed with logarithmic function. Redundancy analysis (RDA) was used to examine water quality and land cover interactions. The canonical correlation indicated the set of variables with the highest explanatory power. This analysis will enable investigation of which landscape factor has the largest influence on water quality (Sliva and Williams 2001).

## Results

### Land cover characteristic

**Fig. 4:**
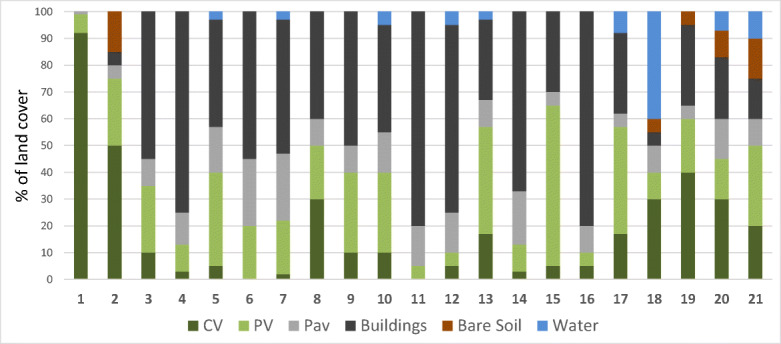
Land use composition within each of 21 patches

A total of 21 patches with 33 sampling sites were classified using the HERCULES model (Fig. 4). It happened that in one patch, there were several hydrological objects; for example, in patch no. 2, there were spring, river and pond sampling sites. There were 6 patches where plant vegetation (FV, CV) covered more than 50% of land and 11 patches where buildings and pavement covered more than 50%. Other patches were characterised by mosaic land cover type with no dominant. Not all features of land cover were extracted in all patches. There were 5 patches found with a small percentage of bare soil and 9 patches with water bodies (this means that surface water such as ponds and rivers accounted for at least 1% of the panel area). The main environmental gradient was an urban development characterised by buildings and pavement.

### Chemical characteristics of water

The water quality parameters varied greatly in space. The lowest water temperature was recorded in springs and the highest in ponds (max temperature 22.4 °C recorded in summer 2014). Spring water was characterised by slight alkalinity (pH = 8.1) compared with the surface water of which pH was 7.7–7.8, on range. Statistically significant differences between water chemistry in springs, streams and ponds were identified for electrolytic conductivity (EC) (*p* < 0.0001), the concentrations of HCO_3_ (*p* < 0.0001), DOC (*p* < 0.0001), nitrate ions (*p* < 0.0001), ammonium ions (*p* = 0.0002) and TN (*p* = 0.0007). SRP varied significantly among water bodies (*p* = 0.003). No statistically significant relationships were found for TP (*p* = 0.0123) and DP (*p* = 0.0101) (Table 5).

**Table 5:** Averages, minima and maxima of water quality parameters. The probabilities associated with Kruskal-Wallis rank sum test are also given (*p* < 0.01)

		Spring			Stream			Pond			*p* value
		*N* = 19			*N* = 81			*N* = 103			
		Average	Min	Max	Average	Min	Max	Average	Min	Max	
Temp	°C	9.3	7.1	13.1	10.7	1.1	22.0	11.8	0.7	24.2	0.5452
pH		8.1	6.5	9.0	7.8	6.4	8.9	7.7	6.3	8.5	0.1838
EC	μS·cm^−1^	595	364	925	838	412	2130	801	235	1469	< 0.0001
HCO_3_^−^	mgC·dm^−3^	69.9	48.8	106.9	65.7	12.4	109.8	57.3	8.2	91.9	< 0.0001
DOC	mgC·dm^−3^	5.9	2.6	34.1	7.2	3.0	22.4	8.9	2.7	17.8	< 0.0001
TN	mgN·dm^−3^	3.40	0.11	9.28	4.23	0.67	13.88	2.67	0.71	7.87	0.0007
NH_4_^+^-N	mgN·dm^−3^	0.11	0.02	0.23	0.30	0.02	0.95	0.23	0.02	0.90	0.0002
NO_2_^−^-N	mgN·dm^−3^	0.003	0.002	0.01	0.007	0.001	0.05	0.004	0.001	0.03	< 0.0001
NO_3_^−^-N	mgN·dm^−3^	0.32	0.03	1.5	1.48	0.05	5.46	3.24	0.02	6.12	< 0.0001
TP	mgP·dm^−3^	0.12	0.03	0.69	0.21	0.02	0.97	0.18	0.05	1.06	0.0130
DP	mgP·dm^−3^	0.052	0.011	0.10	0.09	0.01	0.57	0.09	0.001	0.85	0.0101
SRP	mgP·dm^−3^	0.025	0.003	0.06	0.06	0.001	0.29	0.05	0.001	0.79	0.0030

There are less fluctuations in the chemical parameters of spring water compared with the water of rivers and ponds. Coefficient of variation values indicates low and average variability of pH and HCO_3_^−^ in all types of water. Most of the chemical parameters of water were characterised by high variability. Nitrogen compounds were characterised by greater variability compared with phosphorus compounds in spring water. Extremely high variability of phosphorous compounds was observed in ponds (Table 6).

**Table 6:** Values of the coefficient of variation (CV) for water of springs, rivers and ponds

	Spring	River	Pond
	CV (%)	CV (%)	CV (%)
Temp.	55	57	66
EC	29	42	38
HCO_3_^−^	18	19	40
DOC	44	49	36
TN	68	70	57
NH_4_^+^-N	53	78	78
NO_2_^−^-N	100	110	109
NO_3_^−^-N	105	83	116
TP	49	96	118
DP	41	96	157
SRP	58	99	123

**Fig. 5:**
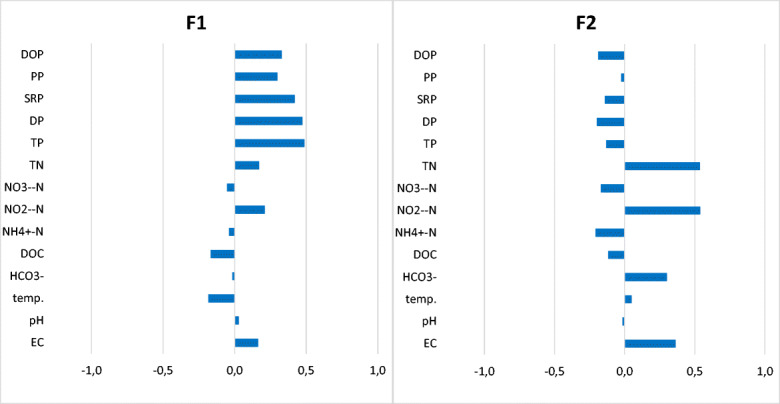
Eigenvectors of individual water quality parameters for factor 1 and factor 2 of PCA analysis

**Fig. 6:**
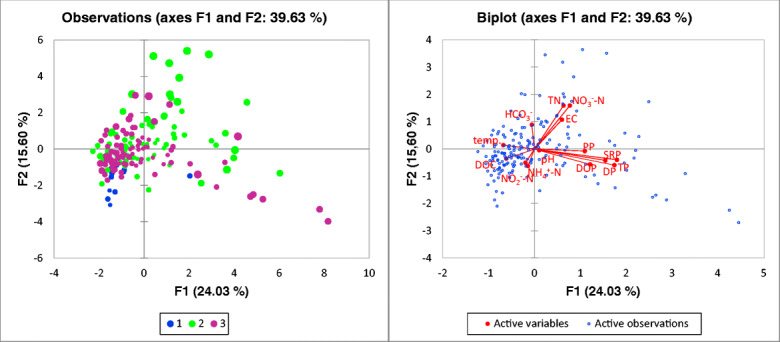
Principal component analysis (PCA) biplot shows water sampling sites (1—spring; 2—river; 3—pond) (*N* = 244) and factor loadings of water quality parameters

PCA analysis revealed clear distinctions between different types of water and water quality parameters. Six principal components were identified with eigenvalues larger than 1. Table 7 presents the eigenvalues, the variance and the cumulative variance. The eigenvectors of individual water quality parameters are shown in Fig. 5. The first component, which explains 24% of the total variance within the dataset, is characterised by high positive loadings for phosphorous compounds and negative loadings for DOC and temperature. The second component, which explains a further 15.6% of the variance, is characterised by high positive loadings for total nitrogen and nitrite nitrogen. The ordination of samples and variables on the PCA biplot is depicted in Fig. 6. Samples from ponds are shown mostly within the left side of biplot, while the right side is occupied mostly by samples from streams. Total nitrogen, total organic nitrogen, nitrate nitrogen and electrolytic conductivity are positively associated with the distribution of water samples along the horizontal axis.

**Table 7:** Eigenvalues, variance and cumulative variance of the principal components of water chemical parameters in surface water and groundwater in Białystok

	Principal component					
	F1	F2	F3	F4	F5	F6
Eigenvalue	3.36	2.18	1.78	1.49	1.28	1.18
Variability (%)	24.03	15.60	12.71	10.67	9.16	8.43
Cumulative (%)	24.03	39.63	52.34	63.01	72.17	80.60

**Fig. 7:**
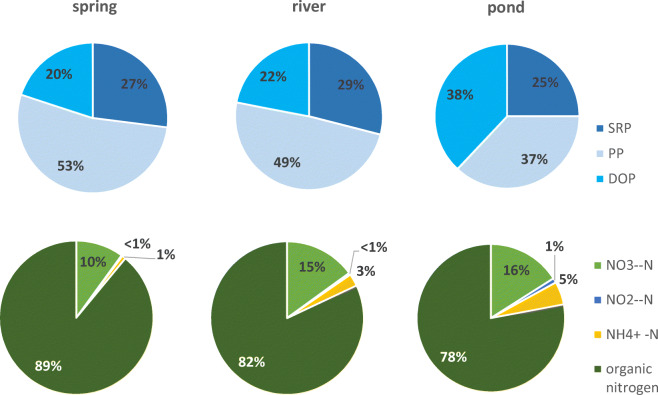
Nitrogen and phosphorous structure of water of spring, river and pond in Dojlidy stream catchment

**Fig. 8:**
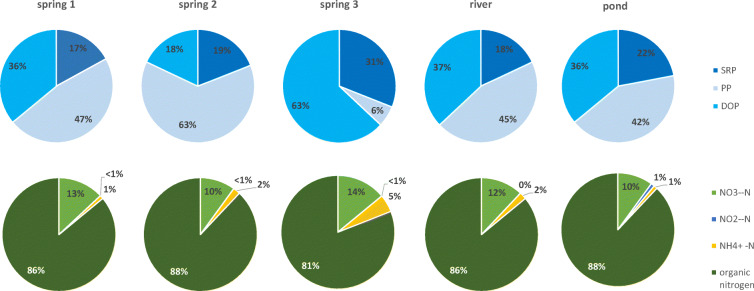
Nitrogen and phosphorous structure of water of spring, river and pond in Jaroszówka catchment

Nutrient concentration analysis in the upper parts of rivers indicates that the structure of phosphorous forms looks different in springs, rivers and ponds. Along the river course, the share of PP decreases, and the share of DOP grows (Fig. 7). Springs in the Jaroszówka catchment are characterised by varying proportions of phosphorus forms. Spring no. 3 (artificial limnocrene) is distinguished by a large proportion of DOP (63%) (Fig. 8). In the case of nitrogen forms, we can see that the organic form dominates in all types of water. Tukey’s test revealed that the DOC concentration in springs was significantly lower than in other types of water (*p* < 0.05) (Tables 8 and 9). No significant differences were found between TP concentrations in the spring, river and pond in the Dojlidy stream catchment (Table 8). Springs in the Jaroszówka catchment were significantly different from each other in terms of TN and nitrate nitrogen concentration (Table 9).

**Table 8:** Water quality characteristic (median value) of spring, river and pond in Dojlidy stream catchment (post hoc Tukey HSD test (*p* < 0.05))

Parameters		Spring	River	Pond	Tukey HSD test, *p* < 0.05
		A	B	C	
TN	mgN·dm^−3^	7.11	5.48	3.21	A–C; B–C
NH_4_^+^-N	mgN·dm^−3^	0.08	0.21	0.14	A–B
NO_2_^−^-N	mgN·dm^−3^	0.002	0.006	0.021	-
NO_3_^−^-N	mgN·dm^−3^	0.64	0.81	0.42	-
TP	mgP·dm^−3^	0.33	0.21	0.13	-
DOC	mgC·dm^−3^	4.2	8.0	9.4	A–B; A–C

**Table 9:** Water quality characteristic (median value) of springs, river and pond in Jaroszówka catchment (post hoc Tukey HSD test (*p* < 0.05))

Parameters		Spring 1	Spring 2	Spring 3	River	Pond	Tukey HSD test, *p* < 0.05
		A	B	C	D	E	
TN	mgN·dm^−3^	11.44	4.38	2.19	5.03	7.21	A–B; A–C; A–D
NH_4_^+^-N	mgN·dm^−3^	0.09	0.08	0.08	0.09	0.08	-
NO_2_^−^-N	mgN·dm^−3^	0.005	0.002	0.003	0.004	0.051	-
NO_3_^−^-N	mgN·dm^−3^	1.42	0.40	0.24	0.59	0.73	A–B; A–C
TP	mgP·dm^−3^	0.24	0.15	0.24	0.21	0.09	-
DOC	mgC·dm^−3^	3.1	4.0	4.4	6.1	7.0	A–D; A–E

### Landscape—water quality linkages

The results from statistical analyses indicated that land cover types were not significantly correlated to many water quality variables in surface water and shallow groundwater in Białystok (Table 10). The temperature had a strong negative relationship with CV. The electrolytic conductivity was negatively related to CV, pavement, buildings and bare soil. Nitrite ions were significantly correlated with the water as a land cover feature. The nitrate ions were positively correlated with pavement and negatively with CV. The phosphorus compounds and DOC were not significantly correlated with any land cover feature (Table 10).

**Table 10:** Results of the Spearman’s rank correlation analysis of water quality variables and land cover features in surface and groundwater in Białystok

Parameters	Land cover features					
	CV	FV	Pav	Buildings	Bare soil	Water
Temp.	− 0.470*	0.172	0.241	0.335	− 0.404	− 0.036
pH	− 0.191	− 0.024	− 0.003	0.094	− 0.227	− 0.405
EC	− 0.517*	− 0.009	0.528*	0.613**	− 0.621**	− 0.282
HCO_3_^−^	− 0.086	− 0.030	0.223	0.294	− 0.224	− 0.472*
DOC	0.151	− 0.224	− 0.045	− 0.163	− 0.183	0.305
TN	− 0.307	0.116	0.393	0.337	− 0.287	− 0.265
NH_4_^+^-N	0.197	0.029	− 0.263	− 0.064	− 0.023	− 0.393
NO_2_^−^-N	0.101	0.056	− 0.153	− 0.007	− 0.041	− 0.569**
NO_3_^−^-N	− 0.494*	0.089	0.475*	0.351	− 0.162	− 0.177
TP	− 0.339	0.364	0.257	0.170	− 0.154	− 0.135
DP	− 0.395	0.349	0.374	0.153	− 0.157	− 0.137
SRP	− 0.413	0.183	0.367	0.163	− 0.107	− 0.259
PP	− 0.211	0.422	0.112	0.083	− 0.063	0.038
DOP	− 0.165	0.391	0.162	0.134	− 0.201	− 0.023

*Significant relationships at a probability level < 0.05

**Significant relationships at a probability level < 0.01

**Fig. 9:**
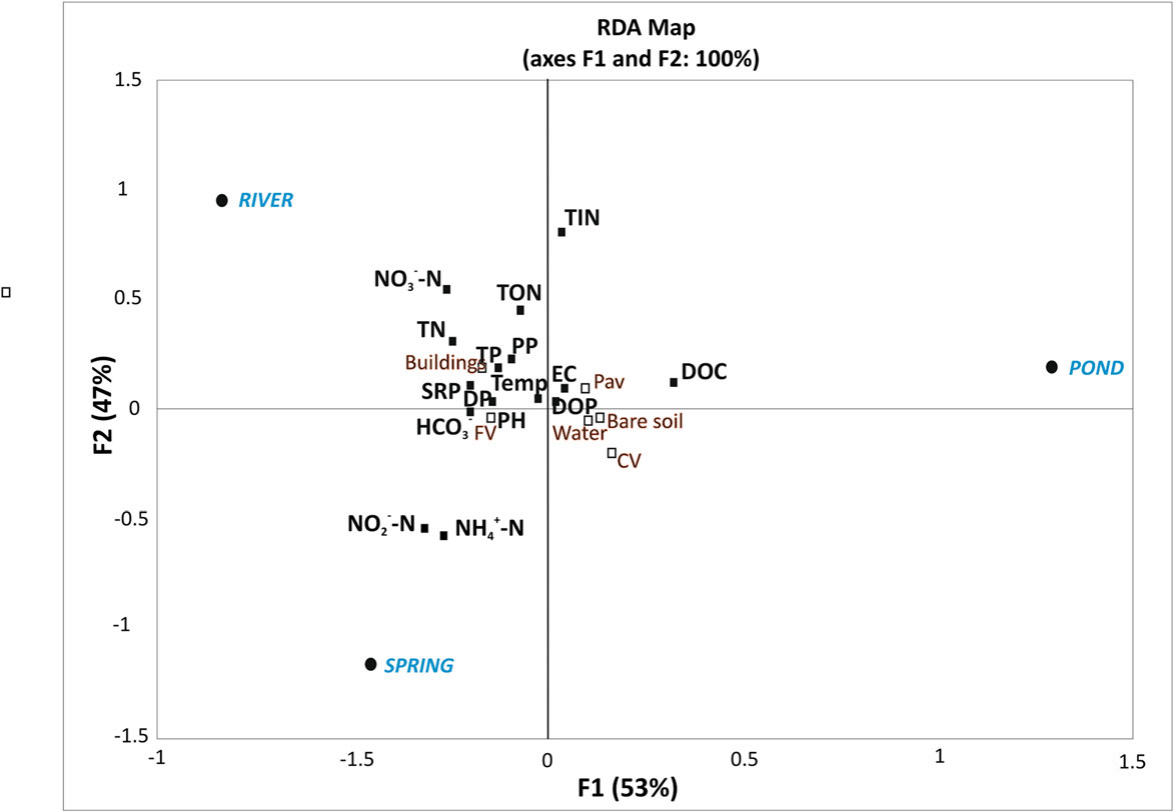
Redundancy analysis (RDA) results using spring, pond and river water quality, and the catchment landscape variables in 21 patches

RDA analysis was carried out to verify which of the selected dominant land covers exerted the greatest impact on the water quality (Fig. 9). All water quality variables were explained by the first two axes (F1 53%; F2 47%). The following variables played important roles: the concentration of nutrients, ammonium ions and DOC. The results of the partial redundancy analysis show that buildings and fine-textured vegetation have the greatest impact on water chemistry (Table 11).

**Table 11:** Percentage of variance accounted for land use. Partitioned by partial redundancy analysis

	Variance component	Explanation (%)
Land cover	CV	23
	FV	25
	Pav	18
	Buildings	30
	Bare soil	2
	Water	2
Total explained		45
Unexplained		55

**Fig. 10:**
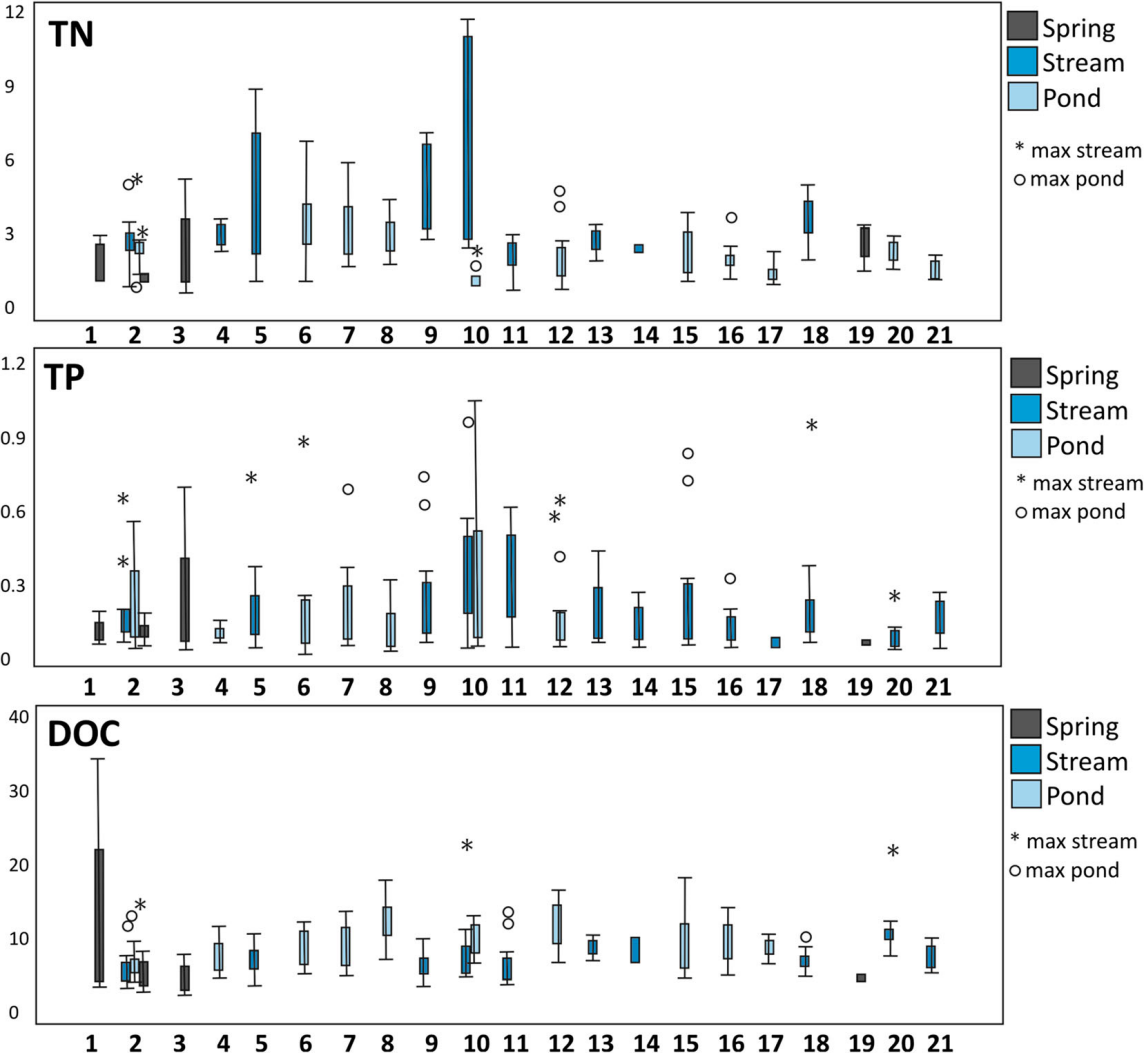
Boxplots representing DOC, TP and TN concentrations from 21 patches

Distribution of nutrients and DOC concentrations were different in each patch. The area analysed in patch no. 10 was characterised by the largest concentration of TN and TP in the river. The patches with buildings and pavements as main land cover features were characterised by the wide gradient of nutrient concentration in rivers and ponds (Fig. 10). The land with forests and shrubs did not have many extreme values in water chemical characteristics. The exception was the distribution of DOC with maximum concentration in forested areas. Shallow groundwater outflows located around the urban centre were rich in organic carbon. Patch no. 1 was characterised by the largest concentration of DOC in shallow groundwater with the maximum value of 34.1 mgC dm^−3^.

## Discussion

Aquatic systems in Białystok are transformed by human activity. Most river valleys are modified, and the water flow is disturbed. However, there are still such areas with little urbanisation pressure. Forested areas in the city constitute a natural enclave, where aquatic ecosystems retain their natural character. Białystok is characterised by the richness of aquatic water bodies (springs, rivers and ponds) which allows us to assess the nutrient cycle in the urban area, including the groundwater component. The locally existing hydraulic connection between groundwater and surface water is a rare phenomenon in an urban area. The quality of groundwater and surface water in terms of concentration of nitrogen, phosphorus and carbon compounds varied. The observed differences were related to anthropogenic transformation, the rate of water circulation and biogeochemical processes. During urbanisation processes, aquatic ecosystems are often used to treat stormwater runoff. This causes a large variation of chemical parameters in surface waters, which is also observed in our research. Surface waters are the main receiver of surface runoff rich in urban contaminations. Our research indicates different vulnerability of various types of waters to pollution. The land cover of the direct catchment of ponds and streams is an important factor affecting water chemistry. Our results show that urban catchment has a significant impact on concentration of nutrients and DOC. Chemical measurements clearly showed differences between springs, streams and ponds. Humans have greatly accelerated nutrients and organic carbon from land to aquatic ecosystems (Vymazal 2010; Wang et al. 2012). This is observed in urban rivers where the concentration of DOC reached the highest value (max 22.4 mgC dm^−3^). Lentic water bodies are exposed to eutrophication, harmful algal blooms and hypoxia. The trophic state of Białystok ponds shows that most of them are hypertrophic and eutrophic (Puczko et al. 2014; Jekatierynczuk-Rudczyk et al. 2016).

Shallow groundwaters are characterised by greater hydrochemical stability than that of rivers and ponds. An important feature of the spring areas is that in winter, spring water does not freeze. Generally, our research shows that springs located in Białystok have a little effect on water quality in heavily urbanised streams. Springs in Białystok are located in a semi-natural environment with forest cover as a natural barrier from urban contamination. In recent years, the increased level of anthropogenic pollution can be found even in groundwater (Banaszuk et al. 2005). The direct interface between aquifer and river, as an important ecotone and as an area of biogeochemical cycling of nutrients and contaminants, is the hyporheic zone (Krause et al. 2011). It is a redox reactive zone where downwelling surface water supplies dissolved oxygen, nutrients and DOC to enable high biogeochemical activity and transformation rates (Krause et al. 2009a, b). Depending on weather, the hyporheic zone may function as a transient source of nutrients (Stofleth et al. 2008). When river flow is undisturbed and hydrometeorological conditions are stable, the hyporheic zone may be one of the primary locations for the processing of nutrients and dissolved and particulate organic carbon (Stubbington et al. 2009). The main characteristics of shallow groundwater, like higher transparency, no suspensions, constant temperature and a generally low content of organic matter, apply to shallow groundwater in Białystok. It is characterised by the lowest average concentration of nutrients and DOC compared with surface water. In contrast, it mostly contains elevated amount of dissolved minerals (Zieliński and Jekatierynczuk-Rudczyk 2010). The land cover impact to water quality in springs was small and similar in each patch. Chemical analyses indicate that electric conductivity of spring water has the lowest value when compared with surface water. Our analyses only show high values of DOC concentration in spring water in polygon no. 1. This results from the location of the spring niche in the forested area with coarse vegetation greater than 90%. Surface runoff causes leaching and transport of organic carbon compounds: humic and fulvic acids in leaf litter and soil (Klimaszczyk and Rzymski 2011). High concentrations of organic carbon are typical of hyporheic or parafluvial waters, which are enriched by DOC from the riparian soils and from interflow, and which contribute significantly to organic matter balance in streams (Krause et al. 2011; Schmidt and Hahn 2012).

This study has demonstrated that water quality in the urban area of Białystok is strongly related to land cover. Application of the HERCULES model enabled spatial analysis of land cover types. The urban space is characterised by a large diversity of landscape elements. Distinction of specific types of land cover in the area of water sampling enabled us to show which land cover variable most affects the water quality parameters. The research shows that the main environmental factors influencing water chemistry are buildings. Buildings create a water-impermeable space where all pollution is rinsed with rainwater. As a consequence, they are an essential element which deteriorates water quality. Scale is an important element affecting relationships between land cover and water quality parameters. In urban areas, at smaller scale, built-up land plays a significant role in influencing nutrient and organic carbon concentration in water (Xiao et al. 2016). The second factor is fine-textured vegetation, which opposite to buildings, has positive influence on water quality. It limits runoff and increases an infiltration surface. Grasslands are an important nitrate source. Nitrogen is mineralised and accumulates in soils during the summer and autumn (Holloway and Dahlgren 2001). Streams flowing through the grassland ecosystems in Białystok tend to have shade-free channels with sand and gravel substrates—conditions favourable for nutrient removal from the water column. This semi-natural ecosystem is a buffer zone that stabilises water runoff and decreases surface runoff.

Despite varied exposure and vulnerability to pollution, our research indicates that the aquatic ecosystems of Białystok are able to cope with the adverse effects of urbanisation. The degree of transformation of the aquatic environment is not big enough, and there is a possibility to improve the quality of water.

## Conclusions

The modelling of hybrid patches using the HERCULES model and combining these patches into dynamic spatial mosaics is a good tool to comprehensively characterise an urban area. Land cover types have an impact on the hydrochemical differentiation of nutrients and organic carbon concentration in water. Water quality of springs, rivers and ponds in Białystok are transformed by urbanisation processes. The degree of transformation is not the same in all types of waters, as evidenced by high concentrations of nitrogen and phosphorus in surface waters. Shallow groundwater has a limited impact on the quality of surface waters because spring water is characterised by better water quality, and modification of river channels in the urbanised area locally limits the hydraulic contact of groundwater with surface waters. In general, most of the contaminations causing the increase of nutrients in water flow come from the surface runoff. Shallow groundwater has a limited effect on surface water quality. Impervious aeration zones, like pavements and buildings, have the most effect on water quality. Trees and shrubs have the function of a buffer zone limiting the surface runoff and flow of nutrients to surface water.
